# Kaiser Permanente Vaccine Study Center: Highlights of 2009–2012

**DOI:** 10.3390/vaccines1020139

**Published:** 2013-04-25

**Authors:** Roger Baxter, Nicola P. Klein

**Affiliations:** Kaiser Permanente Vaccine Study Center, 1 Kaiser Plaza, 16B, Oakland, CA 94612, USA; E-Mail: Nicola.klein@kp.org

**Keywords:** vaccines, research, safety, effectiveness, clinical trials, epidemiology

## Abstract

The Kaiser Permanente Vaccine Study Center is a specialized research organization in Oakland, California. They have been an active vaccine research group for many years, and have participated in and led a multitude of vaccine studies. This article will review the last three years of research activities.

## 1. Introduction

High vaccine coverage is dependent of public perception of vaccine safety. To this end, it is of utmost importance that ongoing research into the potential association of adverse events with immunization be given priority. This article will focus on the activities of a research group devoted to the study of vaccines and vaccine safety.

Founded in 1985, The Kaiser Permanente Vaccine Study Center (KPVSC), part of the Kaiser Permanente Northern California (KPNC) Division of Research (DOR), is a specialized research organization in Oakland, California, which is active in all areas of clinical vaccine research. KPNC is an integrated medical care organization which provides all medical care services to its over 3.2 million members. As part of its core research activities, KPVSC utilizes KPNC member data to conduct a wide range of vaccine effectiveness and safety studies. This article will highlight some of KPVSC’s activities and publications within the past 3 years.

The types of vaccines studies that KPVSC design and carry out can be characterized into six broad categories: vaccine clinical trials; observational vaccine effectiveness studies; active surveillance for vaccine safety, including studies with the CDC-sponsored Vaccine Safety Datalink (VSD); observational post-licensure vaccine safety studies; evaluating vaccine safety in special populations and genetic influences on vaccine adverse events, including studies with the CDC-sponsored and Clinical Immunization Safety Assessment (CISA) Network; and studies evaluating the epidemiology of infectious diseases, vaccine-preventable diseases, and diseases related to vaccine adverse events. 

## 2. Study Highlights

### 2.1. Clinical Trials

KPVSC has a long history of conducting Phase 2 and 3 vaccine clinical trials in infants, children and adults. KPVSC centralizes all research activities in our main Oakland office, with trained KPVSC research nurse and physician sub-investigators located at KPNC clinics throughout the Northern California region. Under the direction of the Principal Investigators and the clinical nurse manager Kathy Ensor, RN, the KPVSC RN and physician sub-investigators have been major contributors or, in some cases, the only site for a number of clinical trials. Since 2009, such trials have included:

GlaxoSmithKline:

1. Evaluated the safety and immunogenicity of a investigational quadrivalent meningococcal vaccine conjugated to tetanus toxoid (MenACWY-TT) in adolescents [1] and infant/toddlers [2]. The United States Food and Drug Administration (FDA) is currently reviewing the adolescent study.
2. Evaluated the safety and immunogenicity of the combination vaccine DTaP-IPV with MMR with and without varicella vaccine in 4–6 year olds [3].
3. Evaluated the safety and immunogenicity of an investigational combination measles, mumps, rubella, varicella (MMRV) vaccine with hepatitis A and 7-valent pneumococcal conjugate vaccine in 1–2 year olds [4].
4. Evaluated the safety and immunogenicity of inactivated hepatitis A vaccine concomitantly with diphtheria-tetanus-acellular pertussis (DTaP) and Haemophilus influenzae type b (HiB) vaccines [5].

Novartis:

1. Safety and immunogenicity of MenACWY—CRM in infants [6], toddlers (with and without MMRV vaccine) [7], children aged 2–10 years [8] and adolescents [9,10]. Novartis’ quadrivalent meningitis vaccine uses a mutant diphtheria toxoid as the conjugate. The product is now licensed for ages 2–55 years in the U.S.
2. Long term follow up studies designed to evaluate the 5 year antibody titer persistence following receipt of MenACWY-CRM in adolescents (P13E1) and infants (P14E1).

Massachusetts Biologics Laboratory:

Study demonstrated the efficacy of one dose of a monoclonal antibody at preventing recurrences of *Clostridium difficile* diarrhea in adults [11,12]. 

Protein Sciences: 

Evaluation of the safety and immunogenicity of a baculovirus derived influenza vaccine (FluBlǾk) in adults aged 50–64 years [13]. This recombinant influenza vaccine has recently been approved by the FDA. 

Pfizer: 

Immune response to Prevnar 13 in adolescents who had previously received Prevnar 7 as infants [14].

Sanofi Pasteur:

Safety and immunogenicity of different multivalent pertussis vaccines in infants and toddlers [15].

### 2.2. Observational Post-Licensure Vaccine Safety Studies

KPVSC is active in conducting Phase IV safety surveillance studies following vaccine licensure, which are typically mandated by the FDA. The size of the studies varies depending on the vaccine, but often range from 10,000 to 100,000 persons vaccinated within KPNC. The studies are observational and evaluate all individuals who received the vaccine as part of routine clinical care. As such, such studies do not involve informed consent; rather, they reflect “real-life” use of vaccines administered to KPNC’s large and diverse population. Using KPNC’s integrated electronic medical record which captures all medical utilization (including vaccinations, laboratory tests, procedures, inpatient, emergency room, and outpatient diagnoses), these studies evaluate post-vaccination outcomes to determine whether such adverse events following immunization (AEFI) are related to the vaccine or whether they are coincidental and unrelated to immunization. In general, our approach to this challenge is to designate a comparison group with which we can compare observed rates of AEFI in the vaccinated group of interest. Comparison groups may include unvaccinated individuals, but vaccinated people can differ in fundamental demographic, health care seeking and other ways from vaccinated individuals [16,17]. It is usually not possible to identify, measure and control for such confounding using data available in electronic medical records, therefore, the results of such studies which rely on unvaccinated controls are difficult to interpret and conclusions drawn may be spurious.

For this reason, we favor utilizing a comparable vaccinated comparison group, such as a group of similarly aged individuals receiving a very similar vaccine during a similar time period. However, if such a comparison group is not readily apparent or available (as is often the case), an alternate approach is to study only individuals who were immunized with the vaccine of interest and to compare the rates of AEFIs during a period of time shortly after vaccination (known as the risk interval) with another period of time either before or after vaccination (known as the control interval).This study design is called the “risk interval” approach. We have utilized this study design in the majority of KPVSC’s ongoing or recently completed post-licensure studies listed below.

GlaxoSmithKline: 

Tetanus, reduced content diphtheria and acellular pertussis vaccine (Tdap, Boostrix). This study evaluated the safety among 10,000 10–18 year olds for selected medical events during the 30 days after vaccination using a risk interval design. It also well as compared outcomes of interest using a historical comparison groups of Td vaccinated teenagers. This study did not detect any safety concerns [18]. 

MedImmune:

Live attenuated intranasal influenza vaccine: LAIV or FluMist^®^ in 5–49 and 2–4 [19,20]. A third publication describes our experience with this live nasal vaccine in children 2–4 years of age [21]. Over multiple years we examined the time periods after immunization, to see if visits to medical clinics, emergency departments, and hospitals were more frequent soon after immunization than at later points in time. We also compared outcomes in those vaccinated with LAIV outcomes in killed injectable influenza vaccine (TIV) and no vaccine. One thing we found is that LAIV recipients are very different from TIV recipients and unvaccinated, and these differences were reflected in utilization patterns. This made interpretation of the results somewhat problematic, and many outcomes needed intense investigation to discern any abnormal pattern. After full investigation, the vaccine appears to be safe for all studied ages, and we found no new areas of concern.

Merck and Co:

1. Human Papilloma Virus (HPV4, Gardasil) vaccine [22,23]: This study evaluated the general safety of HPV4 administered routinely to girls using a risk interval design. Rates for all emergency room and hospitalization events in females who received HPV4 (n = 189,629) were evaluated during the post-vaccination risk interval and compared with rates for the same events during the control period which was a post-vaccination interval distant in time from vaccination. This study found that HPV4 was associated with same-day syncope (OR, 6.0; 95% CI, 3.9–9.2) and skin infections in the 2 weeks after vaccination (OR, 1.8; 95% CI, 1.3–2.4). This study did not detect evidence of new safety concerns among females 9 to 26 years of age secondary to vaccination with HPV [22,23].
2. Zoster vaccine safety [24]: The zoster (shingles) vaccine was approved in 2006 for adults 60 years and older. This study was conducted to monitor the safety of this vaccine when routinely administered to adults and found the vaccine to be very safe. Similar to many post-licensure studies, this study assessed numerous post-vaccination many outcomes, each of which that found a statistical association required follow up and additional investigation. While the study did not identify any safety concerns, interestingly this study found that persons vaccinated with zoster vaccine appeared to be protected against multiple outcomes, including death. Additional analyses revealed that this observation was actually due individuals receiving the vaccine at times when their health was optimized, making it appear (falsely) that the vaccine was protective. In addition, people who were vaccinated appeared to be healthier than the general population of the same age [25].
3. Varicella long term effectiveness and epidemiology after introduction of the vaccine—a 14 year study [26]. Over the 14-year period, we found that Varicella vaccine was very protective (around 90%) against varicella disease without evidence of waning protection. No cases were seen after a second dose. There was no increase of chicken pox in children as they aged, and no increase in zoster (shingles), both of which had been worries prior to the vaccine.

Pfizer:

Prevnar 13 safety: This is an ongoing FDA-mandated study to follow recipients of Prevnar13 in childhood for possible side effects or unexpected adverse outcomes. To date, no significant safety issues have been raised. 

Sanofi Pasteur

1. Tdap (Adacel), in adolescents and adults: The study evaluated the safety of Tdap by evaluating all medical events following immunization of more than 120,000 persons who received Tdap as part of routine clinical care. Final analysis is currently underway.
2. Quadrivalent *Neisseria meningitidis* (ACW135Y) conjugate vaccine (MCV4, Menactra). This study evaluated the safety of this vaccine administered to more than 31,000 individuals ages 11–55 using both observational data in KPNC database (*i.e.*, medical events) and active telephone calls to vaccine recipients. Preliminary analyses using the risk interval method have not detected any safety concerns [27].
3. Menactra in 2–10 year old children. This study will be assessing the safety of this vaccine among a population of 2–10 year old children. Analyses are currently underway.
4. Menactra in 9 and 12 month old children. This study will be assessing the safety of this vaccine among a population of infants and toddlers. Study accrual is underway.
5. Tetanus, diphtheria and acellular pertussis vaccine (DTaP, Daptacel). The primary aim of the study was to assess the general safety of this vaccine in infants. A secondary specific aim focused on the risk of Hypotonic Hyporesponsive Episodes (HHE), a syndrome where infants become transiently less responsive and lose muscle tone. Final analyses are completed. This study identified no safety concerns and no increased risk of HHE related to vaccination.
6. DTaP-inactivated polio-Hemophilus influenza type B vaccines (DTaP-IPV-Hib, Pentacel) safety study. This study is evaluating the general safety of this combination vaccine by evaluating all events in emergency department and inpatient setting and selected outpatient events following immunization of infants and toddlers. Subject accrual has been completed and analyses will begin shortly.

### 2.3. CDC-Sponsored Studies: VSD

The Vaccine Safety Datalink (VSD) is a CDC-sponsored collaboration of 9 medical care organizations throughout the United States (U.S.). VSD’s purpose is to monitor the safety of U.S. licensed vaccines for possible AEFI. Safety evaluations include sequentially monitoring in near real-time either newly licensed vaccines for potential new or unanticipated safety issues following vaccine introduction and widespread use in the U.S. population or if there are changes in the recommendations for already licensed vaccines. Investigators at VSD sites have led or participated in numerous important vaccine safety studies in this collaborative project [28]. KPVSC has led numerous studies in VSD, a number of which are detailed below. 

**Methodologies:** Case centered method, OBS, Exact sequential analyses, and “difference-in-differences”.

*Case Centered Method*: Many vaccine safety studies are designed to answer the question “what adverse events occur after vaccination?” In this method, we reversed this question by and asked “for all individuals who had a specific outcome, did a larger than expected proportion of them receive a vaccine during specific times prior to developing the outcome?” More clustering of vaccines than expected prior to the outcome may suggest that receipt of a vaccine is causally related to the outcome. We look retrospectively at vaccinated people with specific adverse events, and determine what proportion of vaccinations fall in or outside of a risk interval prior to the event. To find our expected odds of vaccination, we determine the proportion of vaccinated people during the same time intervals, for our entire KPNC population, matched by age and sex to the index cases. We have used this approach in multiple studies [16,29,30,31]. 

**Outcome-Based Surveillance (OBS):** This methodological approach extends the principles of the case centered methodology a step further with the goal of monitoring a very large number of vaccine-outcome pairs to preliminarily assess whether a potential relationship exists between the vaccine and the outcome. In order to apply this method, we constructed summary tables of all our members, and eventually of all VSD members, with vaccination, age and sex. This made finding our expected rate of vaccination very efficient, and so we are planning on applying the method to many types of possible adverse events and vaccines, in a project we call Outcome-based surveillance. We expect this project to provide data on various vaccine-AE pairs over years, to be a reference for those looking for potential problems, and to serve as a starting point for vaccine safety research in the years to come. Implementation of this method is ongoing,

**Exact Sequential Analyses (ESA):** This method has been utilized in a number of VSD studies, such as Rapid Cycle Analysis (RCA), where data is analyzed repeatedly over time. ESA is used to adjust for the statistical problem of multiple sequential looks at data. Using an exact permutation test for studies using a binomial outcome, this method improves accuracy, particularly when there are a small number of outcome events. It allows variation in the ratio of the size of comparison groups, and flexibility in controlling for type 1 error over time [32]. Work on a manuscript is ongoing.

Specific Vaccine Safety VSD Studies:

1. Measles-mumps-rubella-varicella combination vaccine and the risk of febrile seizures. This study demonstrated that in 1–2 year old children receiving a first dose, receipt of the measles, mumps, rubella and varicella (MMRV) combination vaccine is associated with a twofold increased risk of febrile seizures 7–10 days after immunization in when compared with separately administered same-day MMR and varicella vaccines [31].
2. Risk of Febrile Seizures Following Measles-Containing Vaccine in 4–6 year old Children. This study evaluated risk of febrile seizures after measles-containing vaccine in 4–6 year old children and no evidence that either MMRV or separately administered MMR and varicella vaccines are associated with an increased risk of febrile seizures in children receiving a second dose of vaccine [33].
3. Effect of age on risk of fever and seizures from measles vaccines: We found that the rate of seizures after measles-containing vaccines is modified by the age of the child receiving the vaccine. Children receiving vaccines on schedule, at one year of age, had a lower rate of seizure compared to those receiving the vaccines later in the second year of life [34].
4. Risk of Rheumatoid Arthritis following vaccination with Tetanus, Influenza and Hepatitis B vaccines among persons 15–59 years of age. This VSD retrospective cohort study assessed for an association between rheumatoid arthritis and 3 different vaccines over a 13 year period. This study did not find any conclusive association between immunization and rheumatoid arthritis [35].
5. Immunization and Bell’s Palsy in Children: A Case-Centered Analysis: Using the Case Centered method described above, this study assessed for an association between vaccinations and the occurrence of Bell’s palsy in children and found no association [29].
6. Lack of association of Guillain Barré Syndrome (GBS) with vaccines [30]: As with the Bell’s palsy study, this study also used the case centered method to examine the risk of GBS after vaccination with any type of vaccine. Evaluating data collected over 13 years which included more than 30 million person-years, we found no association between vaccination and GBS.

### 2.4. CDC-Sponsored Studies: CISA

The CDC-sponsored Clinical Immunization Safety Assessment (CISA) Network is a collaboration between the CDC and multiple academic organizations. The mission of CISA is to assist national efforts to understand the underlying pathophysiology of adverse effects from immunizations, and to provide guidance in diagnosing and managing difficult individual cases where there is a concern about harm from vaccines. KPVSC has been a part of CISA since its inception in 2001 and has been closely involved in a wide variety of CISA activities, including active participation in working groups which provide clinical guidance and expertise to vaccine adverse event clinic consult expertise to address specific patient and medical provider concerns. KPVSC has both led and collaborated on a wide range of CISA studies. Below are a number of KPVSC-led CISA studies.

1. Immunization rates and safety of vaccines administered to children with inborn errors of metabolism (IEM): Using KPNC’s electronic medical record, we identified children with IEM from 1990 to 2007 and assessed immunization rates and AEFI in these children. This study found that children less than 2 years old with IEM in KPNC were not delayed in their receipt of recommended vaccines when compared with healthy infants. This study also did not detect an increased risk for serious adverse event following immunizations among all children 18 years and younger with IEM during 30 days after vaccination [36].
2. Evidence-based approach to defining post-vaccination risk intervals: KPVSC led a CISA working group with the aims of examining how the choice of specific post-vaccination risk intervals (*i.e.*, number of days in the interval, placement of the interval) affect the results of vaccine safety studies. The WG also developed a method by which the best available evidence would be used to determine the “best” risk interval for various adverse events and vaccines. The WG then applied this approach to assess the “best” risk intervals for (1) fever after influenza vaccines and (2) acute disseminated encephalomyelitis after various vaccines [37].
3. Recurrent Guillain-Barré Syndrome Following Vaccination [38]: This study examined all persons with a history of Guillain-Barré syndrome and determined whether receipt of vaccines after GBS diagnosis was associated with a relapse of GBS. This study was reassuring in that in over more than 30 million person-years, there no relapses of GBS after vaccination identified.
4. Recurrent sterile abscesses after vaccination: In this case report, we described two cases of recurrent sterile abscesses which occurred following vaccines containing aluminum adjuvant and discussed a possible association between receipt of vaccines containing higher levels of aluminum adjuvant and development of sterile abscesses [39].
5. Preterm infant responses to Polio vaccine: In this study, we prospectively enrolled 2 month old preterm and term infants and compared their T cell responses to inactivated poliovirus vaccine. The study found that preterm infants develop poliovirus-specific T cell responses that are comparable to those of term infants. However, preterm infants also demonstrated nonspecific and poliovirus-specific functional T cell limitations. Additional studies will be needed to assess whether these deficiencies have clinical implications [40].
6. Risk of fever and sepsis evaluations after immunization in NICU: In this study, we evaluated whether immunization in the neonatal intensive care unit (NICU) were increased after immunizations due to potential post-immunization changes in clinical status. This study did find that there was an increase in fever and cardiorespiratory events after immunization in the NICU, but routine vaccination was not associated with increased risk of receiving sepsis evaluations [41].
7. Vaccination rates at discharge from the NICU: This study determined immunization rates at discharge from the NICU among infants 2 months of age and found that significant proportion of infants *discharged *on or after 2 months of age in the NICU was unimmunized or underimmunized at discharge [42].

### 2.5. Effectiveness Studies

1. Influenza vaccine and mortality in the elderly [16]: Earlier observational studies had shown that influenza vaccine was more effective at preventing death in the elderly than was plausible. We determined that the differences in persons vaccinated with influenza vaccines compared to those not vaccinated were significant, and that many of these differences are not measured by the medical system, so it is not possible to account for their confounding in studies of Influenza vaccine effectiveness (VE). Therefore, in order to bypass this severe confounding, we used a “difference-in-differences” approach, subtracting out the “effects” of vaccination that seemed to occur outside of the influenza season, when no virus is circulating. Although we verified that previous studies had overestimated VE, we demonstrated that the vaccine can still prevent almost half of all deaths attributable to Influenza infection.
2. Flu and hospitalization [43]: Utilizing our same methodology as in the previous study on Flu vaccine and mortality, we determined the effectiveness of influenza vaccination at preventing hospitalization in persons 50 years and older.
3. Pertussis vaccine effectiveness (DTaP waning, Tdap effectiveness, DTaP *vs*. whole cell): In 2010–2011, California experienced the largest outbreak of pertussis (whooping cough) in over 50 years. During the epidemic, pertussis rates markedly increased beginning at age 8 years, peaking at ages 10–11 years, decreasing to age 15 year and were low in adults. We hypothesized that the pattern derived from waning immunity to diphtheria tetanus, acellular pertussis (DTaP) vaccines and undertook several case-control studies in KPNC in which we identified person who tested positive for pertussis and compared them with two different controls: one control group consisted PCR test negative individuals and the other controls were matched individuals identified from the entire KPNC population. Using this approach, we found that (1) the 5th DTaP dose wanes by more than 40% each year after vaccination [44]; (2) Tdap is only about 60% effective at preventing pertussis [45]; (3) the risk of pertussis among teenagers who received 4 doses of whole cell pertussis vaccines during the first 2 years of life was much lower than among teenager who received 4 doses of DTaP [46].

### 2.6. Studies on Epidemiology of Infectious Diseases, Vaccine-Preventable Diseases, and Diseases Related to Vaccine Adverse Events

1. Invasive Pneumococcal disease following introduction of Prevnar 7 and Prevnar 13 into the KPNC population: We have been collecting specimens from patients with invasive pneumococcal infections since 2000 and serotyping them to see whether the serotypes are those that are included in either the 7- or 13-valent conjugate pneumococcal vaccines. Multiple studies are ongoing. Our system allows us to capture data on the entire underlying population, so that incidence rates of disease can be calculated.
2. Bias and flu shots [17]: Studies of influenza vaccine effectiveness are often confounded by unmeasured variables. We showed this in a study looking at multiple years of influenza vaccines. If a person had been vaccinated for all of the preceding 5 years, and then did not get a vaccination in the current year, mortality rose dramatically. However, if another person did NOT get the vaccine for 5 years, and then got vaccinated in the current year, mortality also rose significantly, making it appear that the vaccine both prevents and causes death. This clearly illustrated the problems inherent in retrospective flu studies, and highlighted differences between vaccinated and unvaccinated individuals.
3. Lopsided Windows: Early on in the course of one of our studies noted above [24], we noted that shorter risk time intervals (compared to comparison intervals) and rare rates of events resulted statistically significant differences weighted against the shorter interval. We showed that this phenomenon is predictable, and can happen in any type of study with comparison intervals that vary in size. This finding has been helpful in assessing and interpreting study results [47].
4. Rates of autoimmune disease [48]: This study assessed baseline rates of various autoimmune diseases in KPNC, which is extremely valuable information in both the design and interpretation of vaccine safety studies.
5. Genetics of Staph epidemiology [49]: This study showed that for KPNC, the USA300 clone of MRSA is found both in outpatients and in hospital- and healthcare-acquired settings.
6. Microbiology and epidemiology of skin and soft tissue infections (SSTIs) [50,51,52]: Within our health plan population, we examined the epidemiology of SSTIs, focusing on trends of staphylococcal and methicillin-resistant staphylococcal (MRSA) infections over time. In our health plan, MRSA rates are decreasing over time, though they are still a very important cause of SST
7. Epidemiology of Bell’s palsy [53]: This paper provides a descriptive analysis of the largest population of children with Bell’s palsy to date.
8. Incidence of genital warts among adolescents and young adults prior to HPV vaccination [54].
9. Flu surveillance [55]: We have been monitoring Influenza and Respiratory Syncytial Virus (RSV) infections over many years. We provide a service to the KPNC medical providers and administrators, with weekly updates showing trends and predictions, and including guidelines on testing and treatment. Our publication was on how the H1N1 pandemic threw confusion into the surveillance, because the media releases were very early, before disease really hit our area. We found that fear drove utilization for medical services, even more than real disease. Another study analyzed patterns of flu seasons, and found a recurring pattern of latter seasons each year over 9 seasons [56]. We think that peaks of influenza season are more susceptible to the immunity of the underlying population than to weather, humidity, or other fluctuations in natural conditions.

## 3. Discussion

Vaccines are one of the most important developments in medical history, providing protection from numerous infections, which in the past resulted in considerable morbidity and mortality. Today, those infections have been nearly eradicated in many parts of the world. Predictably, as vaccine-preventable illnesses wane, and parents have little or no knowledge of their severity and impact, concerns of harm from disease turn to fear of harm from vaccines. Many parents are no longer comfortable accepting that vaccines are without or have minimal risk when they are exposed to many other sources (particularly from the internet) that that tell them otherwise. When people go to the doctor for medical care, they are generally looking for something that helps them and their immediate family; appeals to accept vaccines for the benefit of the community are unlikely to motivate them. Although the risk from vaccines is low, they are not completely risk free: there are known adverse effects associated with vaccines, and new associations may yet be discovered. Real or potential risks must be balanced with the benefits of a vaccine, to both the individual and the public at large. The best way to continue benefiting from widespread vaccine coverage is to assure the public that vaccine safety is a high priority for healthcare providers, for the industry that makes vaccines, and for governmental organizations. This reassurance can best be achieved by ongoing surveillance and research into both the safety and the effectiveness of existing and new vaccines. It is crucial for researchers to actively seek out potential problems with vaccines, to quantify adverse events, even when rates are very small, and to determine how well vaccines actually protect against disease, outside of a clinical trial context. In addition, it is important that the medical and scientific literature publish papers on vaccine safety, whether they point out a new issue, or just show once again that vaccines are relatively safe. Research organizations like the Kaiser Permanente Vaccine Study Center are crucial to the study of vaccine safety, and offer a service to vaccine recipients both here in the United States and also the rest of the world. 

## 4. Conclusions

The Kaiser Permanente Vaccine Study Center has been a leader in vaccine studies over many years, and their research has provided answers to a number of questions about vaccine safety and effectiveness. Studies such as these are essential to national efforts to maintain high rates of vaccine coverage. 
